# Factors associated with post-cesarean stillbirth in 12 hospitals in Benin: a cross-sectional

**DOI:** 10.11604/pamj.2016.25.117.9827

**Published:** 2016-10-26

**Authors:** Virginie Mongbo, Edgard-Marius Ouendo, Victoire Agueh, Alphonse Kpozèhouen, Ghislain Sopoh, Jacques Saïzonou, Isabelle Godin

**Affiliations:** 1 Regional Institute of Public Health, University of Abomey-Calavi, BP 384 Ouidah, Benin; 2 Université Libre de Bruxelles. 808 Route de Lennik 1070 Bruxelles, Belgium

**Keywords:** Stillbirth, caesarean section, Benin

## Abstract

**Introduction:**

In spite of free caesarean section applied in Benin since 2009, high rates of stillborn babies continue to be recorded. This study aimed to determine the factors associated with post-caesarean stillborn in Benin.

**Methods:**

Cross-sectional study that covered all women who have delivered by caesarean from December 2013 to February 2014 in twelve hospitals chosen by simple random selection in each of the twelve departments of Benin. Data collected by chart review have been analyzed using the statistical software Epi info 3.5.1. Univariate analysis and multivariable logistic regression were used to identify factors associated with post-caesarean stillbirth at the significance threshold of 5%.

**Results:**

There were 66 stillborn per 1,000 births of which 58% died before admission to hospital. The risk factors identified were the reference (p = 0.0011), general anesthesia (p = 0.0371), the low birth weight (p = 0.0001), the retro-placental hematoma (p = 0.0083), and the umbilical cord prolapse (p = 0.0229). Acute fetal distress (p = 0.0308) and anesthesia administered by an anesthetist nurse or midwife (p = 0.0337) were protective factors.

**Conclusion:**

The majority of cases, in utero death occurred before admission to hospital. Strengthening antenatal refocused consultation, a better access to quality obstetric care and the grant of all obstetric care could reduce stillbirths from caesarean sections in Benin.

## Introduction

Worldwide, each year there are about 2.65 million stillborn in the third trimester of pregnancy, of which 98% in the Low and Middle Income Countries (LMIC) [1]. A higher proportion of these stillborn babies is recorded during childbirth in the LMIC [1, 2], where about half of the pregnancies have reached term, with viable babies if they benefited from better care [1]. The causes associated with stillbirth in LMIC are, among others, the vicious presentation, prolonged labor due to fetal-pelvic disproportion, twin pregnancies. However, fetuses do not die from childbirth labor, but by associated asphyxia, trauma or infection [3]. In developed countries, the impact of these complications is usually reduced by cesarean section [3]. But paradoxically, in many of sub-Saharan African countries, maternal and perinatal mortality following a caesarean section is justified by the low accessibility of women to caesarean and by the poor quality of care [4, 5]. In Benin, cesarean section is made free since 2009 [6] to improve its affordability. But in 2013, as part of data collection from a study on the quality of caesarean section performed in 12 hospitals, 6.6% stillbirth following a caesarean section had caught our attention. As this free caesarean policy aimed at reducing morbidity and infant and maternal mortality and that post-caesarean stillbirth was little published compared to the overall stillbirth [1–3, 7], this study was initiated to describe stillborn babies from the caesarean and to determine their associated factors in Benin.

## Methods

### Study framework

Benin is a country in West Africa divided into 12 departments according to the administrative organization. Its care system is pyramidal, with on top (national level) university-teaching hospitals, at intermediate level (department) department hospitals and at peripheral level, zone (or district) hospitals around which is organized a network of health centers [8]. The study was conducted in the hospitals at departmental and peripheral levels which are reference centers, and carry out the caesarean surgeries. As part of the implementation of free caesarean section, the state gives a package of 100,000 CFA francs (about 153 Euros) to hospitals for each caesarean section performed. This package covers the transfer of the pregnant woman from health center to the hospital (in case of reference) with taking a vein, consultation fees, the cost of the surgical procedure, medicines and medical consumables, the cost of three days of hospitalization for the mother and of the post-surgery control [6, 9].

### Type of study

This was a cross-sectional study conducted from December 18, 2013 to February 8, 2014.

### Study population and sampling

Target selection was made following a two-stage sampling. At the first stage, hospitals have been retained by simple random selection. For financial and time constraints reasons, we opted for the selection of one hospital per department. In each department, the pooling base was the list of all the hospitals at department and peripheral level performing caesarean sections. In total, twelve hospitals have, therefore, been well selected, among which two of intermediate level and ten of peripheral level. At the second stage, we have researched and exploited the records of all women who underwent a caesarean section during the study period in the 12 surveyed hospitals.

### Variables

***Outcome variable:*** the outcome variable was stillbirth. According to the International Classification of Diseases (ICD) Version 10, stillbirth is defined as the death of a fetus weighing at least 1000 grams or of a gestational age of 28 weeks or of a crown length of the heel of 35 cm or more [10]. But since very few stillborn babies are weighed, we considered as stillborn, *an infant born without spontaneous breathing or heart movement* [7], as mentioned in the obstetric records and surgery protocols.

***Independent variables:*** the choice of the independent variables was guided by the data available in the literature [11, 12]. The variables of the database used in this study were: socio-demographic (age, marital status, educational level, place of residence) and gyneco-obstetrical characteristics of the mothers (gravidity, parity, condition of the uterus, gestational age, pregnancy care), medical data (mode of admission, entrance examination, viability (heart sounds) of fetus upon admission, use of the partograph, decision-to-delivery interval, treatment received before caesarean, indications and characteristics of the caesarean section), the characteristics of the baby (stillbirth, state of the stillborn baby (fresh or macerated), weight and presence of malformation). The use of partograph has been appreciated by the availability of the graphic in the records of the women supervised for childbirth labor. Decision-to-delivery interval was defined as the time between the decision and the start of the caesarean section. The caesarean section was considered as programmed when it occurs before the start of birth labor. Otherwise it is emergency.

### Data collection

In each hospital, data were collected by two previously trained midwives who were not from the hospital. Data were collected by review of records, using a sifting form designed from the “Caesarean case Examination“ of the program *Averting Maternal Death and Disability (AMDD)*. But when checking the sifting forms (carried out during supervision by the principal investigator), missing data has been sought in other data supports such as anesthesia register, surgery and childbirth register.

### Data processing

Statistical analyses were performed using the software Epi info version 3.5.1. The description of stillbirths has been done into three groups depending on whether the fetal heart sounds were noticed, not noticed or not documented upon admission of the mother to the hospital. Quantitative variables were expressed by the mean ± standard deviation or median followed by the inter quartile range ([Q1, Q3]) when this variable had an asymmetrical distribution; qualitative variables, per the proportion with a 95% confidence interval. Association between outcome and different independent variables was investigated by univariate analysis, using the Pearson χ2 test or Fisher exact when it was appropriate, at the significance threshold of 5%. The strength of the association has been assessed by the odds ratio (OR) and its confidence interval at 95%. During these crossings, some indications of caesarean section as high blood pressure, other maternal conditions and premature rupture of the membranes were “0” in one of the cells. The multivariate analysis was a “step down” multiple logistic model. We included in the initial model variables from the univariate analysis, which were considered to be associated with the outcome at p < 0.2. Statistical significance to remain in the final multivariable model was set at p < 0.05. The safer modality has been chosen as the reference. The indication of uterine rupture has not been accepted in the model, certainly because of the number of “1” contained in one cell of the contingency table.

### Ethical considerations

The protocol was approved by the Ethics Committee of Research of the Institute of Applied Biomedical Sciences (CER-ISBA), of the University of Abomey-Calavi in Benin.

## Results

Out of 579 women who underwent a caesarean section, 43 perinatal deaths have been registered, among which 38 stillborn, a stillbirth of 66 per 1,000 births, at a median term of 39 (32; 40) weeks of gestation. Mothers of these stillborn were 28.5 ± 6.2 years old and 65.8% were no educated.

### Description of the stillborn babies

Of the 38 stillborn, fetal heart sounds were incurred on the mothers entrance examination in 23.7% (n = 9), not seen in 57.9% (n = 22) and non-documented in 18.4% (n = 7). Subsequently, the description will be made based on these three groups. Table 1 summarizes the characteristics of these stillborn. Among the 6 stillborn with low birth weight, gestational age, documented for 4, was greater than or equal to 37 weeks gestation in 3 cases and lower in one case.

**Table 1:** Description of post-caesarean stillbirths in hospitals of Benin in 2013 (n = 38)

Characteristics	Groups of stillborn according to fetal heart at admission exam		
	Unknown n = 7	No perceived n = 22	Perceived n = 9
**Gestational age (weeks)**			
< 37	1	2	3
≥ 37	4	9	5
Unknown	2	11	1
**Mode of admission**			
Referred from health center	5	22	8
Direct admission	2	0	1
**Type of ceasarean**			
Emergency	7	21	8
Programmed	0	1	1
**Decision-to-delivery interval**			
< 1 hour	1	3	2
≥ 1 hour	6	19	7
**Malformation of the baby**			
**Yes**	0	2	0
No	1	17	6
Unknown	6	3	3
**Stillborn**			
Fresh	2	11	6
Macerated	0	7	0
Unknown	5	4	3
**Birth weight**			
< 2500g	0	2	4
≥ 2500g	7	20	5

### Indications of caesarean

Indications of caesarean section will be described according to each of the three groups of stillborn.

***Stillborn whose fetal heart sounds were collected at admission:*** They all had different indications in simple or double combination: iterative caesarean +retro-placental hematoma, iterative caesarean + pre-uterine rupture, breech presentation + uterine rupture, cross presentation + eclampsia, fetal-pelvic disproportion + acute fetal distress, retro-placental hematoma, iterative caesarean, dynamic dystocia + twin pregnancy. Iterative caesarean concerned a 30-year-old parturient, who once underwent caesarean in the past and who came herself to the hospital at 37 weeks of gestation and was supervised during birth labor. The caesarean section has been determined 2 hours 52 minutes after her admission and performed 14 hours 53 minutes later. Dynamic dystocia was diagnosed in a 17-year-old nullipara, referred at 40 weeks of gestation. Caesarean section occurred 50 minutes after the decision. The moment of admission was not reported. The baby showed no malformation. In the two previous cases, caesarean section was performed under spinal anesthesia by an obstetrician.

***Stillborn whose fetal heart sounds were not perceived on admission:*** The indications in this group were: placenta praevia 3 cases, rupture and pre-uterine rupture 7 cases, maternal rescue 2 cases and one case of the following indications: fetal-pelvic disproportion (contracted pelvis) + acute fetal distress + umbilical cord prolapse , retro- placental hematoma, fetal-pelvic disproportion + uterine rupture, malpresentation + dynamic dystocia, ovular infection + acute fetal distress, malpresentation +umbilical cord prolapse , iterative caesarean + security caesarean, malpresentation, malformation (Siamese twins). Iterative and security caesarean was conducted on unknown term pregnancy on a parturient of 28 years old who previously underwent 2 caesarean sections, showing no other pathology according to her obstetrical record.

***Stillborn whose fetal heart sounds were not documented on admission:*** One case for the following indications has been recorded: fetal-pelvic disproportion (contracted pelvis) + Umbilical cord prolapse, malpresentation + dynamic dystocia, placenta praevia + retro-placental hematoma + malpresentation, placenta praevia + malpresentation + eclampsia, retro-placental hematoma, malpresentation, dynamic dystocia. Dynamic dystocia concerned a 32-year-old parturient, parity 3, who has never undergone a caesarean section, referred at 40 weeks of gestation. Caesarean decided immediately at admission was performed 50 minutes later, under spinal anesthesia by an obstetrician. There was no information neither about birth labor nor on the treatment administered before the decision of caesarean.

### Analytical results: factors associated with stillbirth

They are summarized in Table 2 and Table 3 respectively for the univariate and multivariate analysis. The univariate analysis revealed that age below 25 years, parity below or equal to 4, the previous caesarean section, the acute fetal distress indication were protective factors of stillbirth; and that the low level of education of women, the reference, ambulance transport, emergency caesarean section, general anesthesia, surgical techniques other than that of Pfannentiel and Joel Cohen, the low birth weight, indications for caesarean such as uterine rupture, retro-placental hematoma, placenta praevia and malpresentation expose baby to the risk of stillbirth. The risk factors identified after multivariate analysis were reference, general anesthesia, low birth weight, retro-placental hematoma, and umbilical cord prolapse. Acute fetal distress and anesthesia administered by an anesthetist nurse or midwife were protective factors.

**Table 2:** Univariate analysis: post-caesarean stillbirth in Benin hospitals in 2013 according to the characteristics of the mother, of the caesarean section, of the baby and the indications of caesarean section (n = 579)

Characteristics	Total	Stillborn n (%)	OR	IC 95%	p
**Maternal age (years)**					
< 25	229	8 (3.5)	0.38	[0.17 ; 0.87]	0.0219
≥ 35	72	6 (8.3)	0.96	[0.38 ; 2.45]	0.9355
25- 34	278	24 (8.6)	1	-	
**Educational status**					
No educated	289	25 (8.7)	2.02	[1.01 ; 4.03]	0.0429
Educated	290	13 (4.5)	1	-	
**Parity**	569				
0-4	495	28 (5.7)	0.38	[0.18 ; 0.83]	0.0169
5 et plus	74	10 (13.5)	1	-	
**Mode of admission**					
Referred from health center	363	35 (9.6)	7.58	[3.30 ; 24.94]	0.0001
Direct admission	216	3 (1.4)	1	-	
**Mode of transportation**					
Ambulance	81	10 (12.3)	2.36	[1.10 ; 5.07]	0.0234
Other	498	28 (5.6)	1	-	
**Type of caesarean**					
Emergency	468	36 (7.7)	4.54	[1.08 ; 19.16]	0.0244
Programmed	111	2 (1.8)	1	-	
**Type of anaesthesia**					
General	86	12 (14.0)	2.91	[1.41 ; 6.02]	0.0027
Spinal anesthesia	493	26 (5.3)	1	-	
**Anaesthetist**					
Nurse or midwife	577	37 (6.4)	0.07	[0.00 ; 1.12]	0.1271
Doctor	2	1 (50,0)	1	-	
**Surgical method**					
Others	304	26 (8,6)	2.05	[1.01 ; 4.15]	0.0421
Pfannentiel/Joël Cohen	275	12 (4.4)	1	-	
**Uterine rupture**					
Yes	9	8 (88.9)	144.00	[17.44 ; 1189.12]	0.0000
No	570	30 (5.3)	1	-	
**Retro-placental hematoma**					
Yes	9	5 (55.6)	20.34	[5.21 ; 79.33]	0.0001
No	570	33 (5.8)	1	-	
**Placenta previa**					
Yes	20	5 (25.0)	5.31	[1.81 ; 15.51]	0.0070
No	559	33 (5.9)	1	-	
**Previous cesarean section**					
Yes	164	5 (3.0)	0.36	[0.14 ; 0.95]	0.0318
No	415	33 (8.0)	1	-	
**Acute fetal distress**					
Yes	154	3 (1.9)	0.24	[0.07 ; 0.73]	0.0070
No	425	35 (8.2)	1	-	
**Malpresentation**					
Yes	48	8 (16.7)	3.34	[1.44. 7.77]	0.0088
No	531	30 (5.6)	1	-	
**Umbilical cord prolapse**					
Yes	18	3 (16.7)	3.01	[0.83 ; 10.88]	0.1073
No	561	35 (6.2)	1	-	
**Gestational age (weeks)**	401				
< 37	54	6 (11.1)	2.28	[0.86 ; 6.04]	0.0873
≥ 37	347	18 (5.2)	1	-	
**Birth weight**					
< 2500g	8	6 (75.6)	50.53	[9.81 ; 260.39]	0.0000
≥ 2500g	571	32 (5.6)	1	-	

**Table 3:** Multivariate analysis: factors associated with post-cesarean stillbirth in Benin hospitals in 2013 (n = 579)

Characteristics	OR	IC 95%	p
**Mode of admission**			
Referred from health center	13.63	[2.82 ; 65.76]	0.0011
Direct admission	1		
**Birth weight**			
< 2500g	76.38	[8.58 ; 679.92]	0.0001
≥ 2500g	1		
**Retro-placental hematoma**			
Yes	7.87	[1.70 ; 36.45]	0.0083
No	1		
**Umbilical cord prolapse**			
Yes	5.10	[1.25 ; 20.70]	0.0229
No	1		
**Acute fetal distress**			
Yes	0.25	[0.07 ; 0.88]	0.0308
No	1		
**Type of anaesthesia**			
General	2.43	[1.05 ; 5.62]	0.0371
Spinal anesthesia	1		
**Anaesthetist**			
Nurse or midwife	0.02	[0.00 ; 0.73]	0.0337
Doctor	1		

## Discussion

Our study helped describe and identify factors associated with 38 stillborn post-caesarean in Benin. However, this description is partial due to the missing data. The small number of stillborn could be a limit to the analytical part of the study. Indeed, because of the small number, some variables, even the most suspected as uterine rupture could not be integrated into the logistic regression model. Beyond these limits, the study can be considered as a first exploration of knowledge in this area in Benin. The stillbirth rate in our study is similar to 63 per 1,000 births found by Ouedraogo et al. in Ouidah, Benin [9] but higher than those of several other studies both in developing and developed countries, where stillbirth rate for all deliveries ranged between 7.9 and 32.1 stillborn per 1,000 births [13]. One first explanation for this difference may lie in the definition of stillbirth used. The other explanation may be that, as Ouédraogo et al., we worked only in hospitals and focused on caesarean sections only, while the other studies were focused on deliveries both dystocic and eutocic, and in all types of health facilities.

This hypothesis seems to be confirmed by the identification of the reference as a factor associated with post-caesarean stillbirth. Indeed, if we consider reference as a proxy for severity, 92% of stillborn are from mothers referred from another peripheral healthcare facility. The majority of these cases were either irretrievable (57.9% of babies died in utero, already found on admission to the hospital) or complications such as retro-placental hematoma, umbilical cord prolapse and acute fetal distress, with a poor prognosis for the baby.

However, even if in Benin´s health pyramid, the role of reference center is dedicated to hospitals, the situation of references in this study refers to the delay of decision making in the community, the access to maternity of first level [14] or delay in the administration of the appropriate care in these maternity. Such delays can result from ignorance of the signs of danger by the pregnant women and their families, of a weak financial capacity of the families, a poor availability of qualified human resources in the first-level health facilities or malfunction of the reference system. In fact, out of 35 cases referred from peripheral-level, only 10 (28.6%) were transported by ambulance; the others were on a motorcycle or by taxi. Apart from these transport costs which are in principle covered by the free caesarean policy, the upstream care-taking of the parturient from hospital entails a cost not covered by the policy of free caesarean [6] and could be a problem of affordability and thus justify the second delay.

As a reference center, the hospital should reflect a better quality of care. Unfortunately, mothers whose babies were alive at admission in the hospital, and among whom the indication requires an extreme emergency, have benefitted from this caesarean only after a delay of over one hour, causing therefore the intrapartum death of the babies. While obviously this long response time can be involved, it has not been identified as a factor associated with stillbirth. Similar results had been obtained by Bloom et al. who said that failure to respect the response time is an index of poor quality of obstetrical care [15]. For babies whose death was already noticed on admission, most of the indications for caesarean are justified, except the iterative and security caesarean which the available data didn't allow to justify. Even if saving the mother is a priority, most of these babies were viable (according to their weight and gestational age) and fresh stillborn; they could therefore be also saved if there hadn't been an upstream delay from the hospital.

In the stillborn group where the heart sounds were not documented on admission, the indications seem not all justified. This is the case for example of caesarean section for dynamic dystocia that could have been avoided by non-invasive interventions such as artificial rupture of the membranes, administration of antispasmodics and oxytocin [16]. Unfortunately the lack of information does not allow appreciating the treatment administered before the decision of the caesarean. Some features of cesarean section as the general anesthesia have been identified as factors associated with stillbirth. Indeed, regional anesthesia is the safest and most recommended than general anesthesia, because of less maternal and neonatal morbidity [17, 18]. Ong et al. by comparing newborn children from emergency caesarean section have not observed a neonatal mortality difference but noted that infants born from general anesthesia are more likely to have an Apgar below 7 and to need resuscitation compared to those born by regional anesthesia [19]. Based on these results, and given that of the 38 stillborn babies, 95% were from emergency caesarean sections (so from complicated cases), we can assume that the fetuses were in a precarious clinical condition, which has worsened by the effects of the general anesthesia. The low birth weight could also have exacerbated this effect. As in many other studies, it has been identified as a factor associated with stillbirth [12, 20]. Acute fetal distress identified as a protective factor for stillbirth may be due to a questionable indication, not based on evidence, posing the problem of the quality of care and unnecessary caesareans. To this end, Maaloe et al. have discovered in Tanzania that in 84% of cases of acute fetal distress, fetal heart rate was either comforting or not documented [21].

Finally, our study showed that anesthesia administered by anesthetist nurses and midwives is a protective factor of stillbirth, compared to a doctor anesthetist. This paradoxical result seems to find an explanation in the context of Benin. Indeed, anesthesia in almost all caesarean sections of our study were administered by nurses and midwives, graduated from the National School of Anesthetists Nurses Training of Benin. This is an option of task delegation [22] in order to deal with the lack and poor distribution of doctors anesthesists [23]. In these conditions, we can assume that the doctor anesthesist is solicited only for really complicated or even desperate cases.

## Conclusion

Stillborn after a caesarean in hospitals reflect a poor quality of intrapartum care. In most cases, it is upstream from the hospitals that babies die in utero, suggesting a delay in the community, in the health facilities of first level or malfunction of the reference system. Strengthening refocused antenatal consultation can help reduce the initial delay through a better knowledge of the signs of danger by the pregnant woman and her family. A better access to quality obstetric care, especially prenatal consultation and care during childbirth labor is also essential to reduce stillbirth rate in Benin. Moreover, the free caesarean policy, as implemented almost exclusively in hospitals and focused on caesarean section, will not efficiently reduce stillbirth and thus perinatal mortality. A subsidy of all the care during childbirth could reduce intrapartum complications, late referrals and therefore perinatal and maternal mortality. Meanwhile, we suggest that a quality control mechanism is put in place by the free caesarean agency, and that arrangements are made for the effective implementation of the principles of free policy which help ensure the transportation of parturients referred to the hospital. Meanwhile this reorientation of free policy, we suggest that a quality control mechanism is put in place by the free caesarean agency, and that arrangements be made for the effective implementation of the principles of free policy to ensure the medical transport of parturients referred from health center to the hospital. This would help reduce the post-cesarean stillbirth.

### What is known about this topic

- From the literature it is known that low birth weight, general anesthesia, retro-placental hematoma and umbilical cord prolapse are risk factors for stillbirth.

### What this study adds

- The reference of the woman in labor to a health center to the hospital is a risk factor for post-cesarean stillbirth;
- Acute fetal distress and anesthesia administered by nurses and anesthetists midwives are protective factors of post-cesarean stillbirth in hospitals in Benin.
